# Effects of switching from a high-fat diet to a ketogenic diet on oxidative stress, nitrosative damage, and protein glycation in rat skin

**DOI:** 10.3389/fimmu.2026.1920156

**Published:** 2026-08-27

**Authors:** Natalia Chylińska, Cezary Pawlukianiec, Dominika Malinowska, Małgorzata Żendzian-Piotrowska, Mateusz Maciejczyk

**Affiliations:** 1 Independent Laboratory of Cosmetology, Medical University of Białystok, Bialystok, Poland; 2 Students’ Scientific Club “Biochemistry of Lifestyle Diseases” at the Department of Hygiene, Epidemiology and Environmental Health, Medical University of Białystok, Bialystok, Poland; 3 Department of Hygiene, Epidemiology and Environmental Health, Medical University of Białystok, Białystok, Poland

**Keywords:** ketogenic diet, obesity, oxidative stress, protein glycation, rats

## Abstract

**Background:**

Obesity is a chronic metabolic disease characterized by increased oxidative and nitrosative stress, as well as enhanced protein glycation, all of which adversely affect the skin. Although the ketogenic diet (KD) has been shown to modulate oxidative stress, its effects on skin health remain poorly understood. Therefore, the aim of this study was to evaluate the effects of replacing a high-fat diet (HFD) with the KD on oxidative and nitrosative stress, protein glycation, and extracellular matrix remodeling in rat skin.

**Methods:**

Male Wistar rats were randomly divided into four groups (n=10): standard diet (SD, 14 weeks), SD+KD (8 weeks SD, 6 weeks KD), HFD+SD (8 weeks HFD, 6 weeks SD), and HFD+KD (8 weeks HFD, 6 weeks KD).

**Results:**

Antioxidant concentration and activity did not differ significantly between groups, with the exception of superoxide dismutase (SOD) activity, which was higher in the HFD+SD group compared to the SD and SD+KD groups. The HFD+SD group exhibited higher concentrations of advanced oxidation protein products (AOPPs) compared to the SD group and advanced glycation end products (AGEs) compared to the SD+KD group, as well as increased kynurenine (KN) content compared to the SD and SD+KD groups. Increased levels of crossline-related fluorophores (CRO) were observed in the HFD+SD group compared to the SD+KD group. Matrix metalloproteinase (MMP) activity remained comparable across groups.

**Conclusion:**

Replacing the HFD with the KD may exert beneficial effects on skin metabolism in obese rats. The effects of the KD on skin metabolism appear to be comparable to those of the SD. Further studies are needed to elucidate the effects of the KD on skin metabolism in the context of metabolic diseases.

## 1 Introduction

Obesity is a chronic metabolic disease characterized by excessive accumulation of adipose tissue resulting from a disturbance in the body’s energy homeostasis (1–4). It is considered a major risk factor for many lifestyle diseases, such as type 2 diabetes, metabolic syndrome, and non-alcoholic fatty liver disease (NAFLD), as well as cardiovascular diseases (5–7). The Western diet and the high-fat diet (HFD) are widely recognized as the dietary patterns most strongly associated with the obesity epidemic (8, 9). Diet-induced obesity increases the production of reactive oxygen species (ROS) and reactive nitrogen species (RNS), leading to oxidative and nitrosative protein damage and lipid peroxidation, which in turn results in collagen degradation, accelerated tissue aging, and increased inflammatory responses (10–12). Chronically persistent oxidative stress promotes non-enzymatic protein glycation and the accumulation of advanced glycation end products (AGEs) and advanced oxidation protein products (AOPPs), which further exacerbate degenerative processes (13–15).

As the body’s largest organ, the skin is particularly vulnerable to factors generating oxidative stress (UV radiation, air pollution, stimulants, diet) (16, 17). The skin has its own antioxidant system, encompassing both enzymatic mechanisms: catalase (CAT), superoxide dismutase (SOD), and glutathione peroxidase (GPx), as well as non-enzymatic mechanisms (vitamin C and glutathione (GSH)) (18, 19). Nevertheless, under conditions of increased oxidative and nitrosative stress, its defenses may prove insufficient (17). Consequently, premature skin aging is observed, resulting in wrinkle formation and loss of elasticity (20). ROS also play a significant role in inducing the inflammatory response through the activation of signaling pathways, including nuclear factor kappa B (NF-κB) and the activator protein 1 (AP-1). Activation of NF-κB leads to the overproduction of proinflammatory cytokines such as interleukin -1β (IL-1), interleukin-6 (IL-6), and tumor necrosis factor-alpha (TNF-α), while activation of AP-1 is responsible for collagen degradation (20–23). As a result, chronic inflammation develops in the skin, which promotes the development of inflammatory skin diseases such as psoriasis, acne, and atopic dermatitis (21, 24).

In recent years, there has been increasing interest in the use of various nutritional interventions, such as the ketogenic diet (KD) in patients with obesity (25, 26). The ketogenic diet is a nutritional model in which carbohydrate intake is significantly limited (to 20–50 g per day, which is less than 10% of daily energy requirements), with a high proportion of fat (approximately 70-75% of energy) and a moderate supply of protein (20-25%) (27, 28). This type of nutrition leads to increased production of ketone bodies, changes in energy metabolism, and the body’s transition into a state of ketosis (29, 30). The KD was originally developed as a treatment for drug-resistant epilepsy (31–33). Nowadays, however, it is the subject of numerous scientific studies covering an increasingly broad spectrum of its medical applications, notably metabolic and neurodegenerative disorders, as well as weight loss and improvement of metabolic parameters (34–36). A growing number of scientific reports indicate that the KD may have potential anti-inflammatory and antioxidant effects, including by modulating signaling pathways, including the NOD-like receptor-related inflammasome containing a pyrin domain 3 (NLRP3), nuclear factor erythroid-related factor 2 (Nrf2), and NF-κB, which play a key role in regulating inflammatory processes and oxidative stress (26, 37–40). To date, studies on the effect of the KD on oxidative and nitrosative stress have focused primarily on internal organs such as the liver, muscles, and brain (41–43). Nonetheless, its effect on the skin, especially regarding oxidative and nitrosative stress, glycation processes and extracellular matrix (ECM) remodeling, remains poorly understood (26, 44). Therefore, a better understanding of the effects of switching from the HFD to the KD on skin metabolism may provide new insights into the mechanisms underlying obesity-related skin alterations and contribute to the development of dietary strategies aimed at improving skin health in obesity. The objective of this study was to evaluate the effects of switching from the HFD to the KD on selected markers of oxidative stress, nitrosative stress, and protein glycation in rat skin. We also investigated whether switching from the HFD to the SD would produce comparable effects or whether the KD would be more effective.

## 2 Materials and methods

The study was approved by the Local Ethical Committee for Animal Experiments in Olsztyn (approval no. 9/2023, approval date 18.01.2023).

### 2.1 Animals

The study was conducted at the Center for Experimental Medicine (CMD) of the Medical University of Bialystok, which is certified for compliance with the principles of Good Laboratory Practice (GLP). The experiment was conducted on 4-to-5-week-old male Wistar rats with an initial body weight of 100–120 g. The animals were housed under standard laboratory conditions (22 °C ± 2, 12 h light/12 h dark cycle, relative humidity 55% ± 10%) with free access to food and water. The rats (2–3 per cage) were housed in individually ventilated transparent cages (IVCs) while maintaining visual contact with one another. To ensure animal welfare, the rats were provided with environmental enrichment, including a shelter, gnawing material, and nesting material.

Various animal models of obesity and related metabolic disorders have been described in the literature (45, 46). Obesity can be induced after as little as 5 weeks of HFD feeding, whereas longer feeding periods may additionally lead to insulin resistance and/or hyperglycemia (47, 48). Therefore, an 8-week HFD feeding period was selected for the present study (49, 50). The duration of KD feeding in this model was also determined based on the available literature (49, 50).

**Figure 1:**
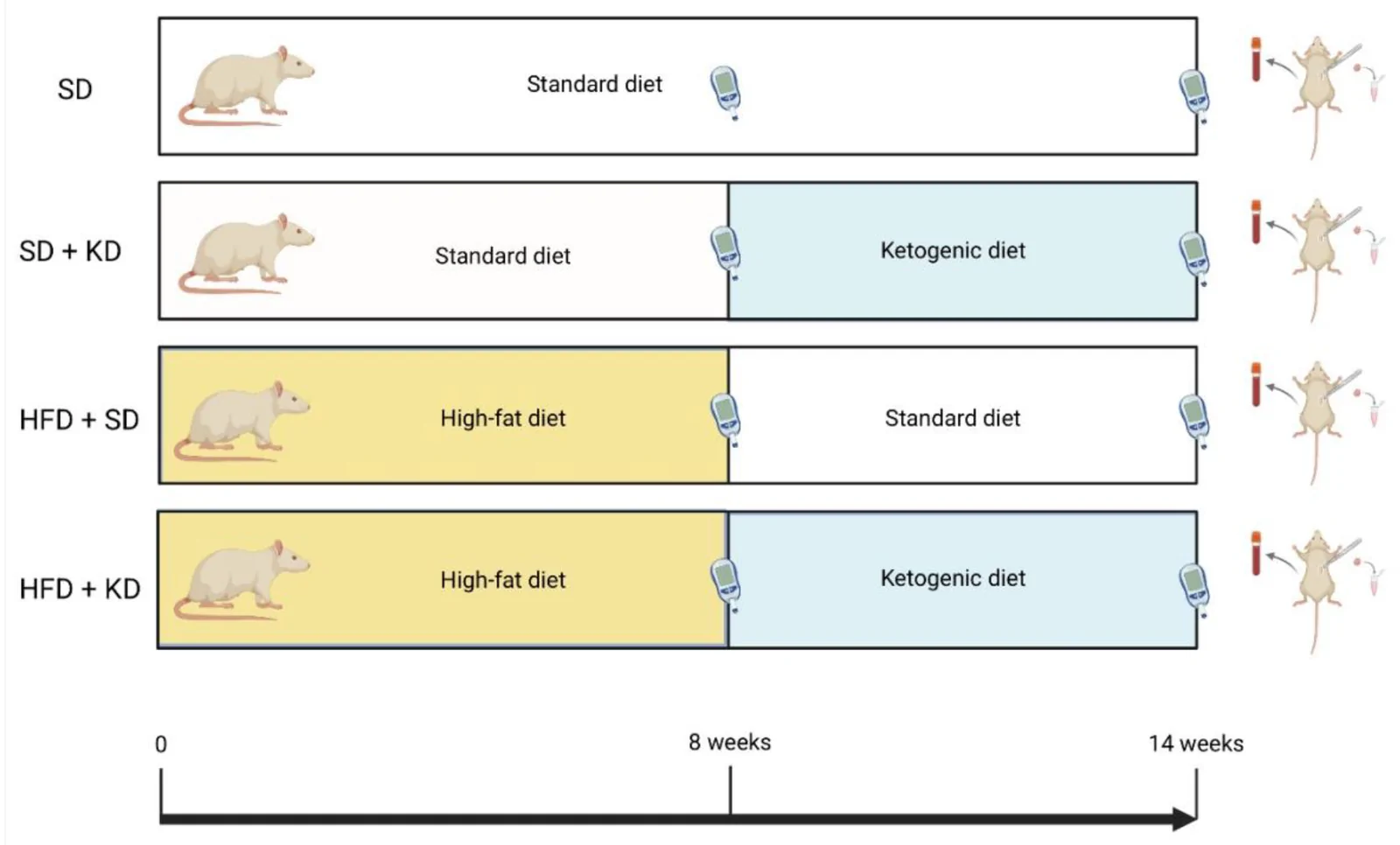
Experimental groups. Created using biorender.com. SD, standard diet; KD, ketogenic diet; HFD, high-fat diet.

After a 7-day acclimatization period, the rats were randomly assigned to four groups (n = 10 per group):

- Control group (SD): animals were fed a standard diet for 14 weeks (Ssniff, Soest, Germany; 9% kcal from fat, 24% kcal from protein, 67% kcal from carbohydrates).
- SD + KD group: animals were fed a standard diet for 8 weeks followed by a ketogenic diet for 6 weeks (Altromin C1084; 84% kcal from fat, 5% kcal from carbohydrates, 11% kcal from protein).
- HFD + SD group: animals were fed a high-fat diet for 8 weeks (Research Diets Inc., D12492; 60% kcal from fat, 20% kcal from carbohydrates, 20% kcal from protein), followed by a standard diet for 6 weeks.
- HFD + KD group: animals were fed a high-fat diet for 8 weeks followed by a ketogenic diet for 6 weeks (**Table 1**, **Figure 1**).

**Table 1:** Energy density and macronutrient composition of experimental diets.

Nutritional parameter	Unit	SD	KD	HFD
Energy density	cal/g	3225	6431	5240
Protein	(% of total energy)	24	11	20
Carbohydrate	(% of total energy)	67	5	20
Fat	(% of total energy)	9	84	60
Saturated fat	(% of total fat)	17.6	32	34.3
Monounsaturated fat	(% of total fat)	19.8	35.9	22.5
Polyunsaturated fat	(% of total fat)	62.6	32	43.3

SD, standard diet; KD, ketogenic diet; HFD, high-fat diet.

Body weight was recorded weekly throughout the experimental period.

After 8 weeks of dietary intervention (standard diet or high-fat diet), fasting blood glucose levels were measured from tail vein blood using a strip glucometer (Accu-Chek, Bayer, Germany) to confirm the development of hyperglycemia in HFD-fed animals.

### 2.2 Euthanasia and sample collection

At the end of the dietary intervention, the rats were fasted for 12 h and anesthetized intraperitoneally with sodium pentobarbital (40mg/kg body weight) (51, 52). Once complete loss of reflexes was confirmed, the animals were euthanized by blood withdrawal from the abdominal artery. Skin tissue samples (approximately 2 × 2 cm) were collected immediately, rinsed with cold physiological saline, blotted dry, weighed, snap-frozen in liquid nitrogen, and stored at -80 °C until biochemical analysis.

Skin homogenates were prepared by mechanical homogenization in ice-cold phosphate-buffered saline (PBS, pH 7.4), followed by centrifugation at 10,000×g for 15 min at 4 °C (52). The resulting supernatants were used for subsequent determinations of oxidative stress, nitrosative damage, protein glycation markers and ECM remodeling.

### 2.3 Assessment of systemic glucose metabolism and insulin resistance

Fasting blood glucose concentrations were measured in whole blood collected from the tail vein after a 12 h overnight fast using a handheld glucometer (Accu-Chek, Roche Diagnostics, Germany), as previously described in experimental models of diet-induced insulin resistance (53, 54). Plasma insulin levels were determined using a commercially available rat/mouse insulin ELISA kit (cat. no. EZRMI-13K, Merck Millipore, USA), according to the manufacturer’s instructions. Absorbance was measured at 450 nm with wavelength correction at 590 nm using a microplate reader, and insulin concentrations were calculated from a standard calibration curve.

Insulin resistance was estimated using the homeostasis model assessment of insulin resistance (HOMA-IR), calculated according to the following formula: HOMA-IR = [fasting glucose (mmol/L) × fasting insulin (µU/mL)]/22.5, as originally proposed and validated for rodent models (55).

### 2.4 Determination of lipid profile, free fatty acids, and ketone bodies

Plasma free fatty acid (FFA) concentrations were quantified using a colorimetric Free Fatty Acid Quantitation Kit (cat. no. MAK044, Sigma-Aldrich, USA), following the manufacturer’s protocol (56). Triglyceride (TAG) levels were determined using a Triglyceride Quantification Colorimetric/Fluorometric Assay Kit (cat. no. MAK564, Sigma-Aldrich, USA), with absorbance measured at 570 nm, as previously described in experimental metabolic studies (57). Total cholesterol was assessed using a Cholesterol Assay Kit (cat. no. ab65390, Abcam, UK).

Plasma ketone body levels were evaluated by measuring β-hydroxybutyrate concentrations using a β-Hydroxybutyrate Assay Kit (cat. no. MAK041, Sigma-Aldrich, USA), a widely applied method for assessing nutritional ketosis in rodent models (58). All lipid and ketone body measurements were performed in accordance with the manufacturers’ instructions, and concentrations were calculated using standard curves supplied with each assay.

### 2.5 Determination of liver and renal function markers

Plasma activities of alanine aminotransferase (ALT, EC 2.6.1.2), aspartate aminotransferase (AST, EC 2.6.1.1), and γ-glutamyl transferase (GGTP, EC 2.3.2.2), as well as concentrations of creatinine, urea, and uric acid, were determined using enzymatic colorimetric assays with commercially available diagnostic reagents (BioSystems S.A., Barcelona, Spain). Measurements were performed according to the manufacturer’s instructions.

Plasma ALT and AST activities were assessed using kinetic methods, which monitor the rate of NADH oxidation during the enzymatic transamination reactions catalyzed by aminotransferases. The decrease in absorbance was recorded spectrophotometrically at 340 nm, and enzyme activities were expressed in units per liter (U/L).

Plasma GGTP activity was determined using a kinetic colorimetric assay based on the transfer of the γ-glutamyl group from a synthetic donor substrate to glycylglycine, forming a chromogenic product measurable spectrophotometrically at 405 nm.

Plasma creatinine concentration was determined using an enzymatic colorimetric method in which creatinine is converted through a series of enzymatic reactions to generate hydrogen peroxide, which subsequently reacts with a chromogenic substrate to form a colored compound measurable at a wavelength of 500 nm.

Plasma urea concentration was measured using the urease–glutamate dehydrogenase enzymatic method. In this assay, urease hydrolyzes urea to ammonia and carbon dioxide, and the produced ammonia is subsequently utilized in a glutamate dehydrogenase reaction coupled with NADH oxidation, monitored spectrophotometrically at 340 nm.

Plasma uric acid levels were quantified using an enzymatic colorimetric assay based on uricase-mediated oxidation of uric acid to allantoin and hydrogen peroxide. The generated hydrogen peroxide reacts with chromogenic substrates in the presence of peroxidase, producing a colored compound detectable spectrophotometrically at 520 nm.

### 2.6 Non-enzymatic and enzymatic antioxidants

Skin ascorbic acid (AA) was assessed using the colorimetric method of Jagota and Dani, in which ascorbate reduces the Folin reagent, producing a colored product detectable at 760 nm (59). Both parameters were expressed relative to the total protein concentration. Skin reduced glutathione (GSH) was determined spectrophotometrically using Ellman’s method, based on the reaction of thiol groups with DTNB, yielding a chromophore measurable at 412 nm (60).

Skin superoxide dismutase (SOD, EC 1.15.1.1) activity was assessed according to Misra and Fridovich, based on the inhibition of adrenaline auto-oxidation to adrenochrome, monitored at 480 nm; one unit of SOD activity was defined as the amount of enzyme required to inhibit adrenaline oxidation by 50% (61). Skin catalase (CAT, EC 1.11.1.6) activity was determined spectrophotometrically by measuring the rate of hydrogen peroxide (H_2_O_2_) decomposition at 240 nm, following the method described by Aebi, and expressed as units per milligram of protein, where one unit represents the amount of enzyme decomposing 1 µmol of H_2_O_2_ per minute (62).

### 2.7 Total oxidative balance

Skin total antioxidant capacity (TAC) and total oxidant status (TOS) were assessed using validated colorimetric methods reflecting the reducing and oxidizing potential of samples (63). TAC absorbance was read at 660 nm, and TOS at 530 nm. The oxidative stress index (OSI) was calculated as: OSI = (TOS/TAC) × 100. All parameters were normalized to protein content.

### 2.8 Lipid peroxidation and protein oxidation

Skin malondialdehyde (MDA) and 4-hydroxynonenal (4-HNE) concentrations were determined using the 1-methyl-2-phenylindole (1-MPI) method described by Gérard-Monnier et al. (64). Absorbance of the chromophore was read at 586 nm, and values were calculated from tetraethoxypropane standards.

Skin total thiols (TT) were quantified using Ellman’s reagent (DTNB) (60). Absorbance of TNB was measured at 412 nm. Protein carbonyls (PC) were measured using the 2,4-DNPH spectrophotometric method (65), with absorbance at 355 nm. Results were expressed as nmol/mg protein.

### 2.9 Protein glycation

Skin Amadori products (AP) were quantified colorimetrically using the nitro blue tetrazolium (NBT) assay, in which early glycation intermediates reduce NBT to a formazan chromophore measurable at 525 nm (66). Amyloid-like structures (Amyloid) were assessed fluorometrically using thioflavin T, which binds β-sheet–rich aggregated protein conformations typical of advanced glycation; fluorescence was recorded at Ex/Em = 385/485 nm (67).

To further characterize glycation pathways, several fluorescent glycation products were measured at their characteristic excitation/emission wavelengths. These included arginine-derived glycation products (ARG) (320/380 nm), carboxymethylarginine/crossline-related fluorescent species (CRO) (380/440 nm), vesperlysines-type fluorophores (VES) (350/405 nm) and pentosidine (PEN) (335/385 nm) (68). Together, they reflect complementary stages of protein glycation and glycoxidation, ranging from early modifications to advanced cross-linking structures.

Skin total advanced glycation end-products (AGE) were assessed by recording intrinsic AGE fluorescence at Ex/Em = 370/440 nm in samples diluted 1:5 with PBS. All glycation parameters were expressed as arbitrary fluorescence units (AFU) per milligram of protein.

### 2.10 Protein glycoxidation

Skin dityrosine (DT) and N-formylkynurenine (NFK) were determined as fluorescence-based markers of oxidative modifications of tyrosine and tryptophan residues. Both analytes were measured according to the protocol described by Hawkins et al. (69). Prior to analysis, samples were diluted in 0.1 M sulfuric acid (1:5, v/v), after which fluorescence emission was recorded at 330/415 nm for DT and 325/434 nm for NFK. All fluorescence signals were normalized to total protein content to ensure comparability across groups.

Skin kynurenine (KN) was measured fluorometrically. Samples were diluted with 0.1 M sulfuric acid in a 1:5 (v/v) ratio, and fluorescence was recorded at 365/480 (69). Results were standardized to the fluorescence of 0.1 mg/mL quinine sulfate in 0.1 M sulfuric acid measured under identical conditions. Final values were expressed relative to total protein content.

Skin advanced oxidation protein products (AOPPs) were measured spectrophotometrically. Samples were diluted 1:5 (v/v) in PBS, placed in a 96-well microplate, and mixed with potassium iodide and acetic acid. Absorbance was read immediately at 340 nm and quantified using chloramine-T standards (70).

### 2.11 Nitrosative damage

Skin peroxynitrite was quantified using an ELISA method, following established methods used for detecting nitrative markers in tissues. 3-nitrotyrosine (3-NT) was determined using a commercial diagnostic kit (cat. no. K 7824, Immundiagnostik AG; Bensheim, Germany). Both results were normalized to protein content.

### 2.12 Apoptosis markers

Skin caspase-3 (Cas-3, EC 3.4.22.56) activity was measured using a colorimetric method with Ac-Asp-Glu-Val-Asp p-nitroanilide as the substrate. Following enzymatic cleavage, the release of p-nitroaniline was quantified spectrophotometrically at 405 nm (71). Caspase-9 (Cas-9, EC 3.4.22.62) activity was determined analogously using Ac-Leu-Glu-His-Asp p-nitroanilide as the substrate, and absorbance of p-nitroaniline was measured at 405 nm (72). Enzyme activities were expressed as pmol of p-nitroaniline released per minute per milligram of protein.

### 2.13 Signaling pathways associated with oxidative stress and extracellular matrix remodeling

Skin nuclear factor erythroid 2-related factor 2 (Nrf2) and nuclear factor kappa B (NF-κB) were determined using commercially available enzyme-linked immunosorbent assay (ELISA) kits according to the manufacturers’ protocols. Nrf2 concentrations were assessed using the Rat Nuclear Factor Erythroid 2-Related Factor 2 ELISA Kit (cat. no. E3093r, EIAab, Wuhan EIAab Science Co., China), whereas NF-κB levels were measured using the Rat NF-κB ELISA Kit (cat. no. ER1186, FineTest, Wuhan Fine Biotech Co., China). Absorbance was recorded with a microplate reader at a wavelength of 450 nm, and the obtained values were normalized to total protein content.

The activity of skin matrix metalloproteinases MMP-1 (EC 3.4.24.7), MMP-2 (EC 3.4.24.24), MMP-9 (EC 3.4.24.35) and MMP-11 (EC 3.4.24.B3) were determined fluorometrically using the synthetic fluorogenic substrate MCA-Pro-Leu-Gly-Leu-Dpa(Dnp)-Ala-Arg-NH_2_ (10 µM), where MCA represents the fluorescent group (7-methoxycoumarin-4-yl) acetate and Dnp acts as a quencher. The assay is based on enzymatic cleavage of the substrate by active metalloproteinases, resulting in increased fluorescence intensity proportional to enzyme activity. Prior to activity measurements, samples were incubated with 1 mM 4-aminophenylmercuric acetate to activate pro-MMPs for 3 h for MMP-1, 1 h for MMP-2 and 2 h for MMP-9, while MMP-11 was analyzed in its active form without prior activation. Fluorescence was measured at excitation/emission wavelengths of 325/393 nm according to previously described protocols (73, 74).

### 2.14 Statistical analysis

Statistical analyses were conducted using GraphPad Prism 10.4.2 (GraphPad Software, San Diego, CA, USA). The distribution of each variable was assessed with the Shapiro-Wilk test. As most datasets did not meet the assumptions of normality, results are presented as median values with minimum and maximum ranges, and non-parametric procedures were applied. Overall differences among the four experimental groups (SD, SD+KD, HFD+SD, HFD+KD) were examined using the Kruskal-Wallis one-way analysis of variance on ranks, followed by Dunn’s multiple comparisons test for *post hoc* analyses. Wilcoxon signed-rank test was used for before-and-after comparisons. Multiplicity-adjusted *p-values* are reported, and *p < 0.05* was considered statistically significant.

The sample size was determined *a priori* based on previous experiments (75). A significance level (α) of 0.05 and a statistical power of 80% (β = 0.20) were assumed. The minimum required sample size was seven rats per group; however, ten rats were included in each group.

## 3 Results

### 3.1 General characteristics

**Figure 2:**
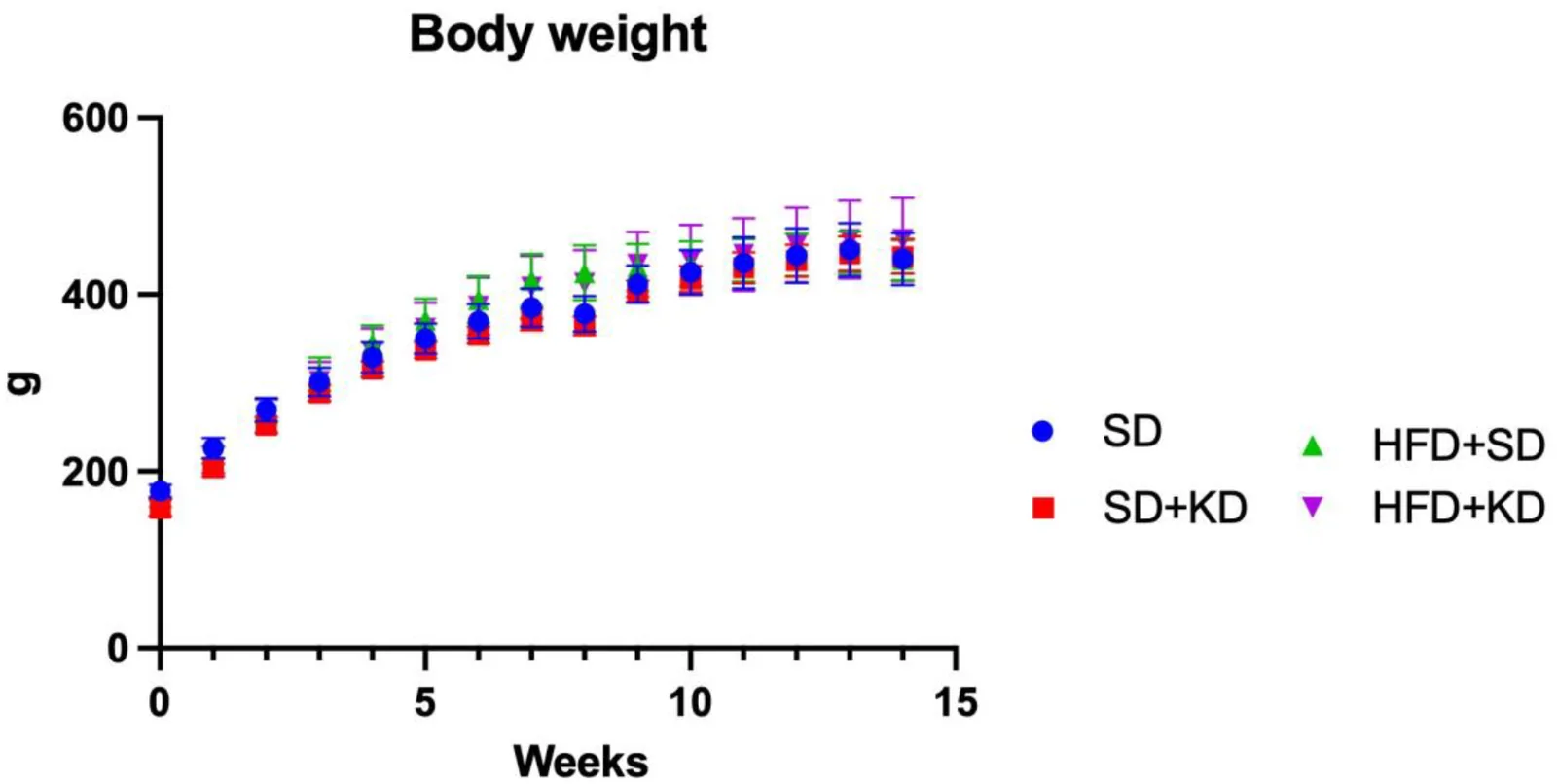
Body weight of rats. The results are presented as the median, minimum, maximum, and percentiles. HFD, high-fat diet; KD, ketogenic diet; SD, standard diet.

At baseline, no significant differences in body weight were observed among the four experimental groups (SD, SD+KD, HFD+SD, HFD+KD), confirming appropriate randomization prior to dietary intervention (**Figure 2**, **Table 2**).

**Table 2:** General characteristics of rats.

Parameter	SD	SD+KD	HFD+SD	HFD+KD	p-value SD+KD vs. SD	p-value HFD+SD vs. SD	p-value HFD+KD vs. SD	p-value HFD+SD vs. SD+KD	p-value HFD+KD vs. SD+KD	p-value HFD+KD vs. HFD+SD
Baseline										
Body weight [g]	121.8 (106.0-136.3)	110.0 (100.0-120.0)	116.0 (110.0-120.0)	114.0 (108.0-124.0)	0.2544	>0.9999	>0.9999	0.8017	>0.9999	>0.9999
Week 8										
Body weight [g]	379.6 (339.3-410.7)	368.0 (342.2-378.3)	431.0 (371.3-450.6)	429.6 (357.3-457.0)	>0.9999	0.2048	0.2436	0.0069	0.0068	>0.9999
Fasting glucose [mg/dL]	104.0 (83.0-126.0)	99.5 (84.0-121.0)	181.0 (152.0-205.0)	177.0 (131.0-203.0)	>0.9999	0.0152	0.0403	0.0012	0.0041	>0.9999
End of the experiment										
Body weight [g]	447.4 (395.7-481.2)	443.4 (408.0-479.7)	445.4 (403.6-465.3)	460.4 (405.4-539.7)	>0.9999	>0.9999	>0.9999	>0.9999	>0.9999	>0.9999
Body weight gain [g]	64.45 (35.17-80.96)	83.24 (52.56-101.4)	20.00 (-5.130-32.84)	48.34 (30.80-82.74)	0.7725	0.0178	>0.9999	<0.0001	0.0880	0.1643
Food intake [g/day/rat]	24.25 (21.06-26.61)	23.77 (19.64-25.27)	15.02 (13.50-15.98)	14.63 (12.33-15.63)	>0.9999	<0.0001	<0.0001	0.0003	<0.0001	>0.9999
Fasting glucose [mg/dL]	99.00 (87.00-133.0)	92.00 (84.00-108.0)	122.0 (93.00-134.0)	102.0 (94.00-115.0)	0.9447	0.4874	>0.9999	0.0096	0.5448	0.6991
Insulin [μIU/mL]	24.84 (10.32-33.96)	17.90 (12.99-30.27)	19.57 (8.640-29.99)	17.63 (6.900-25.16)	>0.9999	>0.9999	0.9330	>0.9999	>0.9999	>0.9999
HOMA-IR	5.040 (2.420-6.520)	3.190 (2.850-4.290)	4.720 (3.860-7.080)	4.635 (3.050-5.930)	>0.9999	>0.9999	>0.9999	0.4589	0.6554	>0.9999
TAG [mg/dL]	76.76 (69.21-79.48)	66.55 (51.56-73.26)	73.99 (68.07-75.19)	71.58 (65.98-75.29)	0.0010	0.7440	0.1334	0.3101	0.8383	>0.9999
Total cholesterol [mg/dL]	67.76 (63.12-69.88)	67.96 (61.96-69.32)	68.81 (63.46-71.07)	68.56 (57.78-75.78)	>0.9999	>0.9999	>0.9999	>0.9999	>0.9999	>0.9999
FFA [mmol/L]	0.28 (0.22-0.35)	0.14 (0.10-0.17)	0.21 (0.15-0.30)	0.13 (0.10-0.23)	0.0013	0.4978	0.0017	0.3424	>0.9999	0.3947
β-hydroxybutyrate [ng/uL]	51.10 (39.35-55.41)	60.93 (55.31-80.92)	56.93 (54.41-60.76)	60.36 (56.40-68.05)	0.0034	0.2638	0.0034	0.9171	>0.9999	0.9171
ALT [U/L]	36.37 (11.5-61.58)	50.16 (3.92-153.2)	35.5 (7.92-78.58)	54.79 (36.5-80.74)	0.9119	>0.9999	0.1605	>0.9999	>0.9999	>0.9999
AST [U/L]	47.37 (29.83-79.41)	59.58 (25.00-131.3)	47.58 (36.58-72.58)	49.20 (36.33-65.41)	>0.9999	>0.9999	>0.9999	>0.9999	>0.9999	>0.9999
GGTP [U/L]	4.444 (1.481-7.407)	6.481 (3.703-11.11)	5.555 (4.074-6.296)	7.407 (5.925-11.48)	0.0563	>0.9999	0.0007	0.5773	>0.9999	0.0377
Creatinine [mg/dL]	0.3411 (0.2078-0.6522)	0.3189 (0.1633-0.4744)	0.1189 (0.03000-0.2078)	0.1189 (0.03000-0.2522)	>0.9999	0.0077	0.0055	0.0236	0.0214	>0.9999
Urea [mg/dL]	35.56 (25.68-48.85)	24.46 (11.82-29.96)	33.87 (28.54-36.48)	24.97 (12.55-30.54)	0.0076	>0.9999	0.0066	0.0393	>0.9999	0.0398
Uric acid [mg/dL]	2.488 (0.6829-7.415)	1.561 (1.220-2.293)	1.805 (0.4390-2.244)	1.927 (1.220-2.634)	0.0493	0.5218	>0.9999	>0.9999	0.7558	>0.9999

ALT, alanine aminotransferase; AST, aspartate aminotransferase; FFA, free fatty acids; GGTP, γ-glutamyl transferase; HFD, high-fat diet; HOMA-IR, homeostasis model assessment of insulin resistance; KD, ketogenic diet; SD, standard diet; TAG, triglycerides. The results are presented as the median (minimum-maximum).

After 8 weeks of feeding, body weight was significantly higher in both HFD-fed groups compared with SD+KD animals (+17% in HFD+SD, +17% in HFD+KD), whereas no significant differences were observed relative to SD controls. In contrast, fasting glucose levels were significantly elevated in both HFD-fed groups relative to SD controls (+74% in HFD+SD, +70% in HFD+KD), as well as relative to SD+KD animals (+82%, +78%, respectively), confirming successful induction of obesity and hyperglycemia at this stage of the experiment. No significant differences were observed between HFD+SD and HFD+KD (**Figure 2**, **Table 2**).

At the end of the experiment, final body weight remained comparable across all groups (**Figure 2**, **Table 2**).

Food intake was significantly lower in both HFD groups compared with SD animals (-38% in HFD+SD, -40% in HFD+KD). Additionally, food intake was also significantly reduced in HFD-fed rats compared with SD+KD animals (-37% in HFD+SD, -39% in HFD+KD) (**Table 2**).

Body weight gain was significantly reduced in HFD+SD rats compared with SD controls (-69%) and compared with SD+KD animals (-76%). No significant differences in body weight gain were detected in the remaining pairwise comparisons (**Table 2**).

Regarding glucose metabolism at the end of the experiment, fasting glucose did not differ significantly between groups relative to SD controls. Notwithstanding, HFD+SD rats exhibited higher fasting glucose compared with SD+KD animals (+33%). Plasma insulin concentrations and HOMA-IR values remained comparable across all groups (**Table 2**).

Triglyceride concentrations were significantly reduced in the SD+KD group compared with SD animals (-13%). Free fatty acid levels were significantly decreased in SD+KD and HFD+KD relative to SD controls (-50%, -54%, respectively). β-hydroxybutyrate concentrations were significantly increased in both ketogenic groups compared with SD animals (+19% in SD+KD, +18% in HFD+KD) (**Table 2**).

Total cholesterol as well as ALT and AST activities did not differ significantly between groups. However, GGTP activity was significantly elevated in HFD+KD compared with SD controls (+67%) and compared with HFD+SD animals (+33%) (**Table 2**).

Markers of renal function showed several significant differences. Creatinine levels were significantly lower in both HFD-fed groups compared with SD controls (-65% in HFD+SD and -65% in HFD+KD) and compared with SD+KD animals (-63% and -63%, respectively). Urea concentrations were significantly reduced in SD+KD and HFD+KD relative to SD controls (-31%, -30%, respectively); additionally, urea was higher in HFD+SD compared with SD+KD (+38%) and lower in HFD+KD compared with HFD+SD (-26%). Uric acid levels were significantly reduced in SD+KD compared with SD animals (-37%), while no other comparisons reached statistical significance (**Table 2**).

### 3.2 Non-enzymatic and enzymatic antioxidants

**Figure 3:**
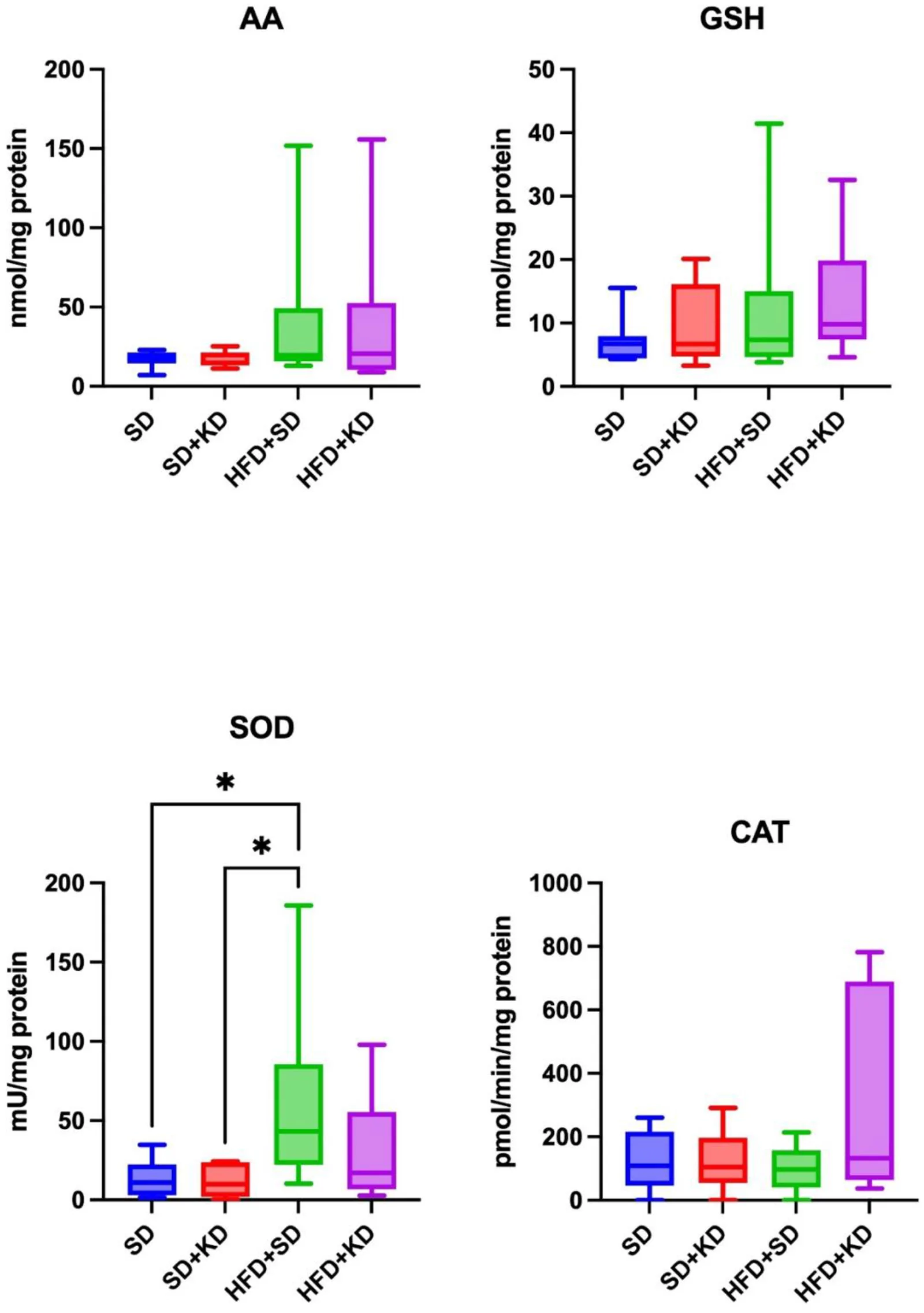
Concentration of non-enzymatic and enzymatic antioxidant markers in the skin of rats. The results are presented as the median, minimum, maximum, and percentiles. AA, ascorbic acid; CAT, catalase; GSH, reduced glutathione; HFD, high-fat diet; KD, ketogenic diet; SD, standard diet; SOD, superoxide dismutase *p < 0.05.

The concentrations of non-enzymatic antioxidants, including AA and GSH, did not differ significantly between any of the experimental groups. Among enzymatic antioxidants, SOD activity showed statistically significant variation, with the HFD+SD group displaying higher SOD activity compared with both the SD (+295%, p = 0.0219) and SD+KD (+336%, p = 0.0182) groups. No other comparisons reached statistical significance, and CAT activity remained unchanged across groups (**Figure 3**).

### 3.3 Total oxidative balance

**Figure 4:**
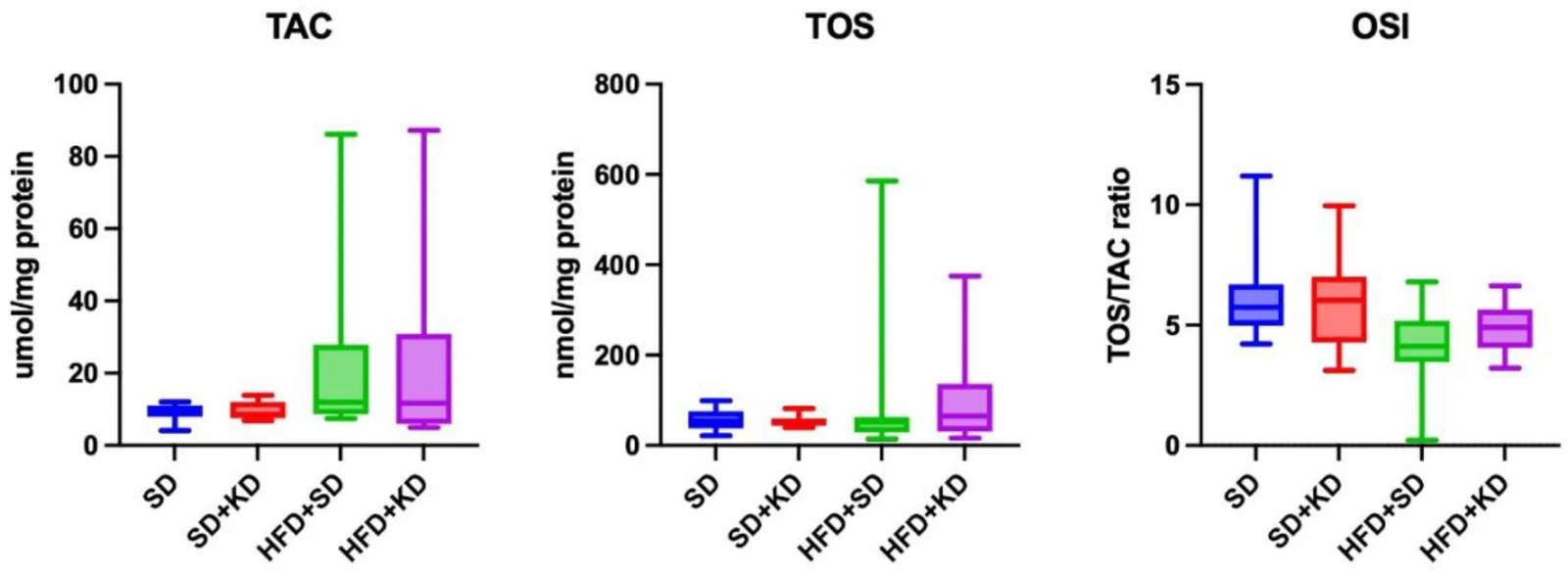
The oxidative stress index and concentration of TAC and TOS in the skin of rats. The results are presented as the median, minimum, maximum, and percentiles. HFD, high-fat diet; KD, ketogenic diet; OSI, oxidative stress index; SD, standard diet; TAC, Total antioxidant capacity; TOS, total oxidant status.

Total antioxidant capacity (TAC), total oxidant status (TOS), and the resulting oxidative stress index (OSI) did not differ significantly among the four dietary groups (SD, SD+KD, HFD+SD, HFD+KD) (**Figure 4**).

### 3.4 Lipid peroxidation and protein oxidation

**Figure 5:**
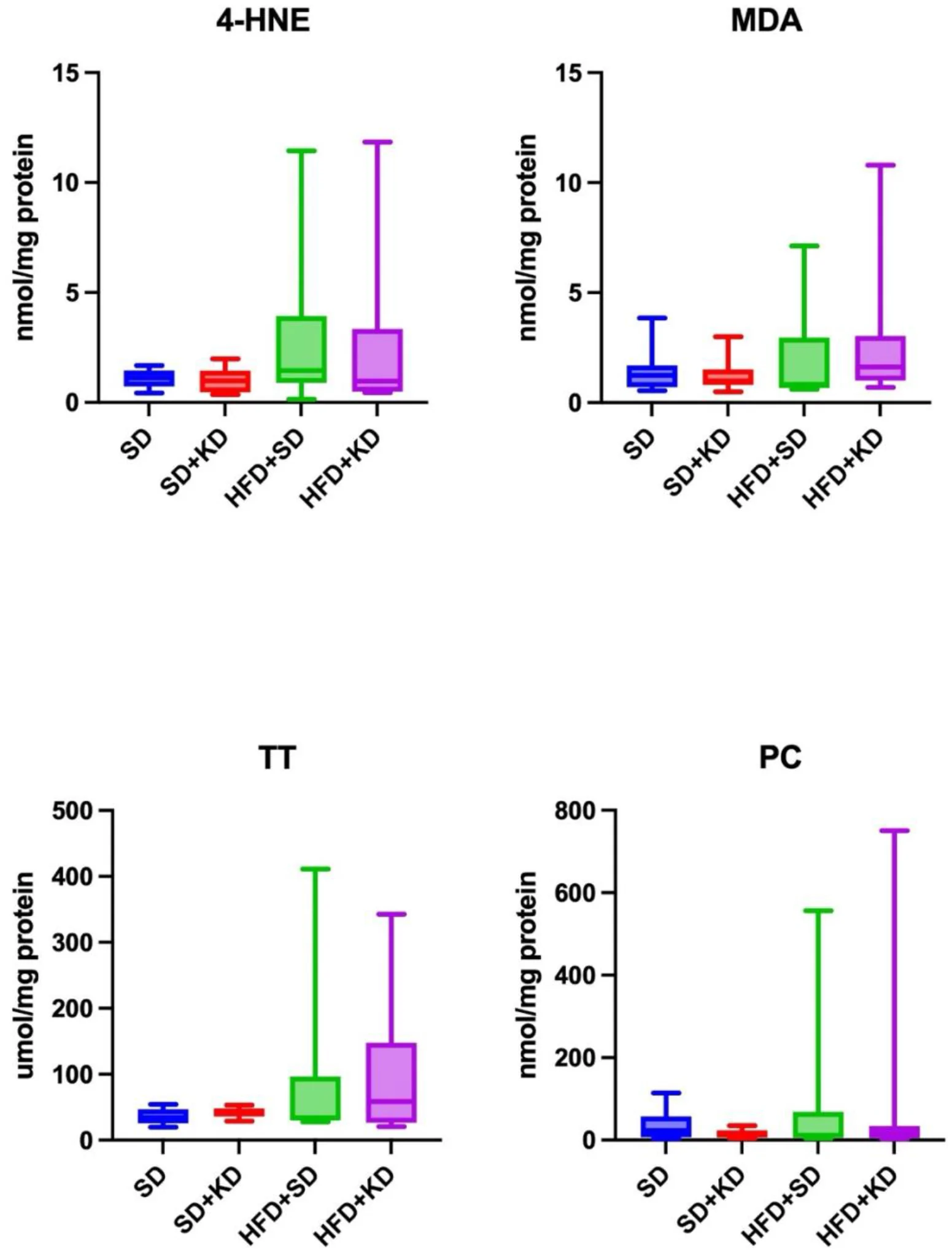
Concentration of lipid peroxidation and protein oxidation markers in the skin of rats. The results are presented as the median, minimum, maximum, and percentiles. 4-HNE, 4-hydroxynonenal; HFD, high-fat diet; KD, ketogenic diet; MDA, malondialdehyde; PC, protein carbonyls; SD, standard diet; TT, total thiols.

The concentrations of malondialdehyde (MDA) and 4-hydroxynonenal (4-HNE), as well as total thiols (TT) and protein carbonyls (PC), did not differ significantly between the experimental groups. All measured parameters remained statistically comparable across SD, SD+KD, HFD+SD, and HFD+KD (**Figure 5**).

### 3.5 Protein glycation

**Figure 6:**
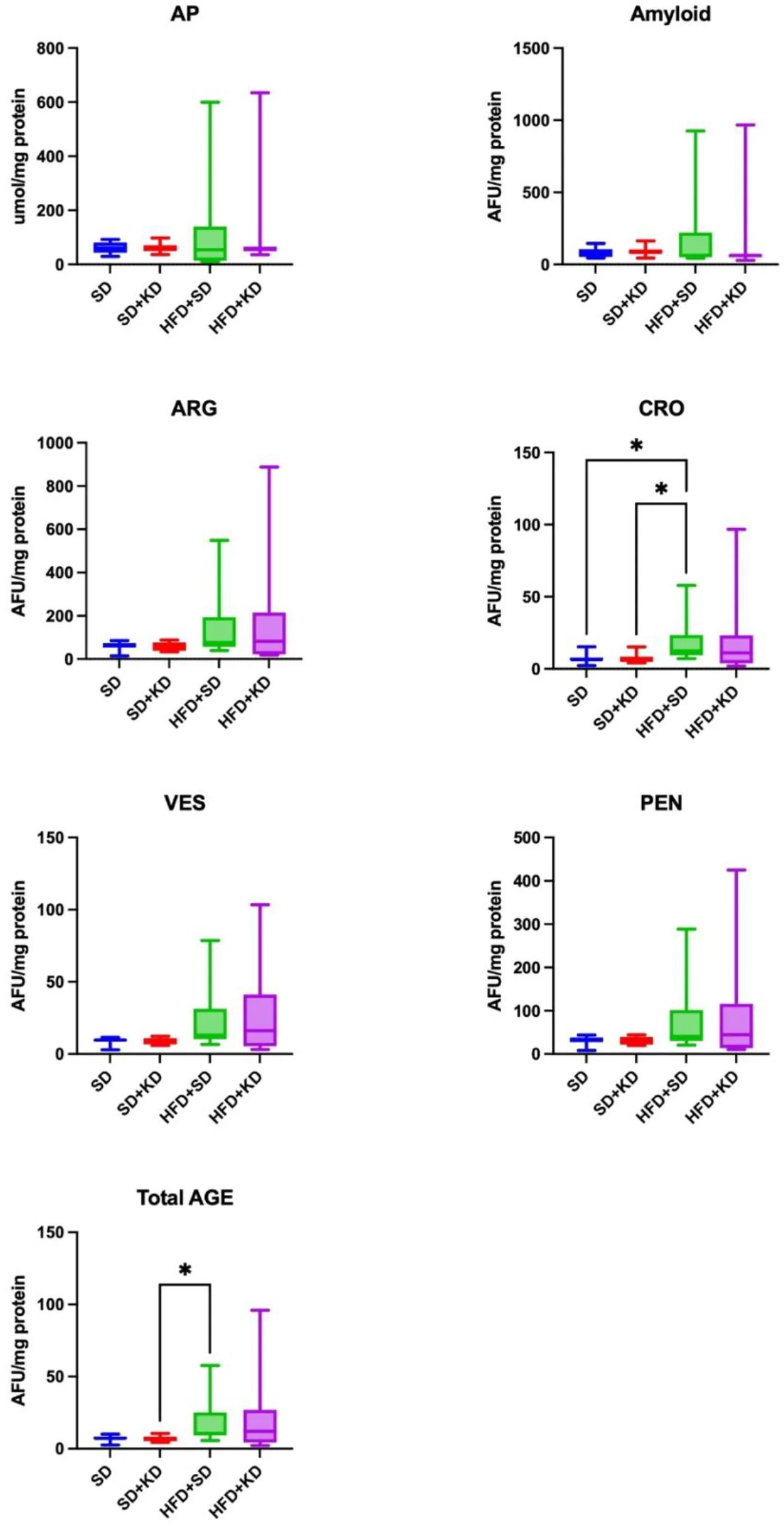
Concentration of protein glycation markers in the skin of rats. The results are presented as the median, minimum, maximum, and percentiles. AGE, advanced glycation end-products; Amyloid, amyloid-like structures; AP, Amadori products; ARG, arginine-derived fluorescence; CRO, carboxymethylarginine/crossline-related fluorophores; HFD, high-fat diet; KD, ketogenic diet; PEN, pentosidine; SD, standard diet; VES, vesperlysines. *p < 0.05.

CRO levels were higher in the HFD+SD group compared with both the SD (+180%, p = 0.0420) and SD+KD groups (+177%, p = 0.0374). Total AGE levels were also elevated in the HFD+SD group relative to the SD+KD group (+185%, p = 0.0058). No other comparisons reached statistical significance. No statistically significant differences were found for AP, Amyloid, ARG, VES, or PEN across the experimental groups (**Figure 6**).

### 3.6 Protein glycoxidation

**Figure 7:**
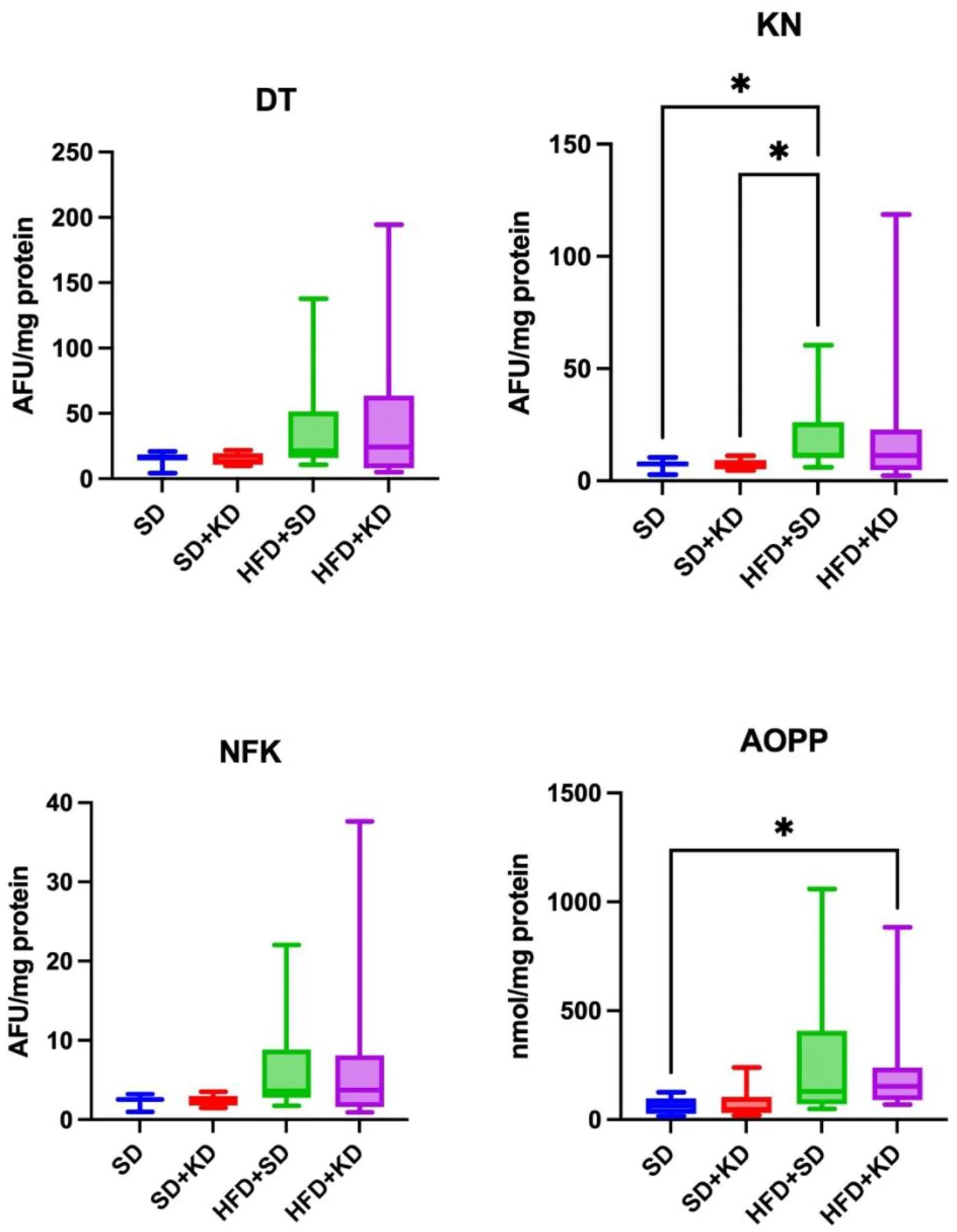
Concentration of protein glycoxidation markers in the skin of rats. The results are presented as the median, minimum, maximum, and percentiles. AOPP, advanced oxidation protein products; DT, dityrosine; HFD, high-fat diet; KD, ketogenic diet; KN, kynurenine; NFK, N-formylkynurenine; SD, standard diet. *p < 0.05.

In contrast, KN exhibited significant variation, with the HFD+SD group showing higher KN levels compared with both the SD group (+184%, p = 0.00396) and the SD+KD group (+174%, p = 0.00420). No other comparisons reached statistical significance. AOPP concentrations were also significantly altered, with higher levels observed in the HFD+SD group compared with the SD group (+350%, p = 0.00420). DT and NFK did not show statistically significant differences between any of the experimental groups (**Figure 7**).

### 3.7 Nitrosative damage

**Figure 8:**
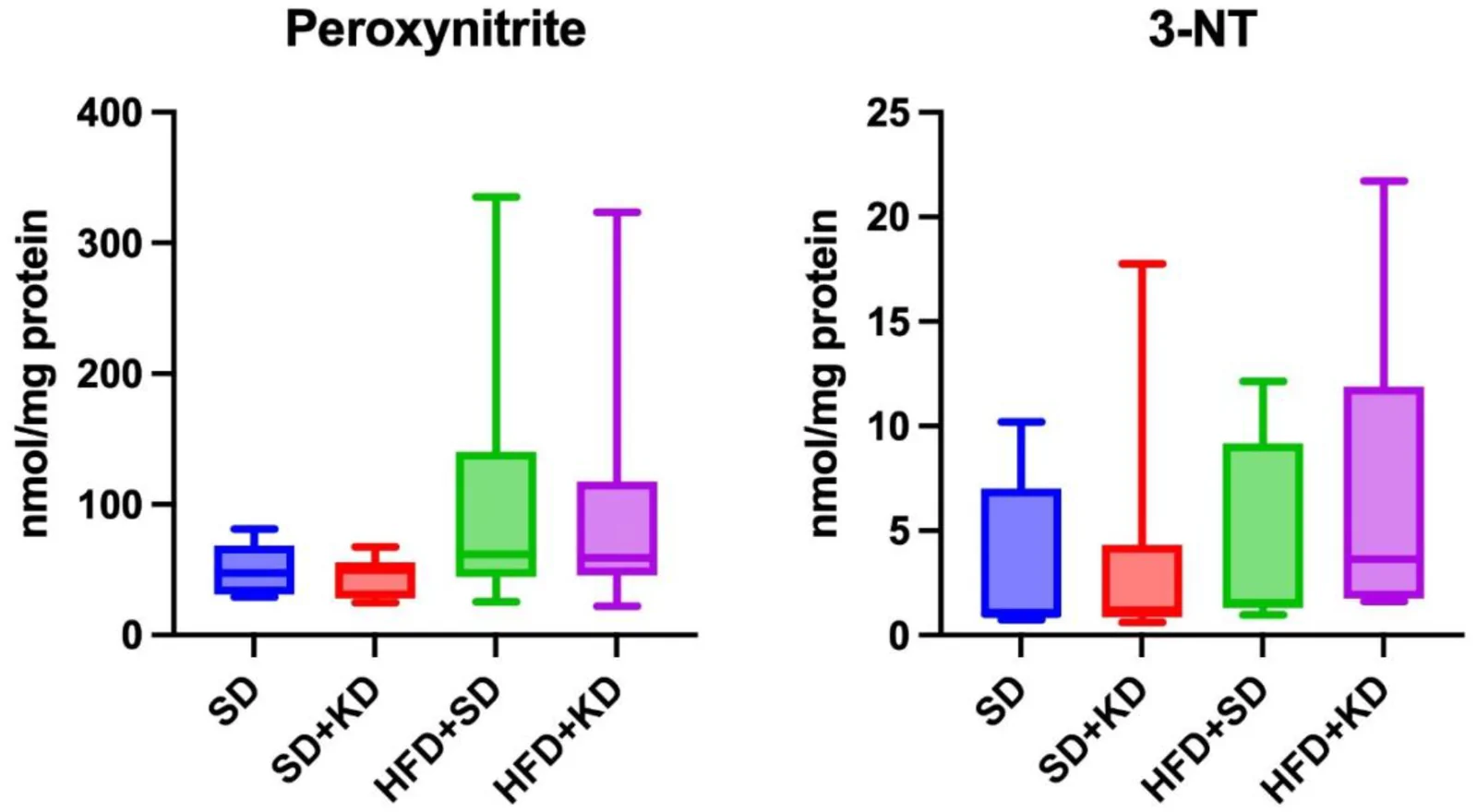
Concentration of nitrosative damage markers in the skin of rats. The results are presented as the median, minimum, maximum, and percentiles. 3-NT, 3-nitrotyrosine; HFD, high-fat diet; KD, ketogenic diet; ONOO^−^, peroxynitrite; SD, standard diet.

Neither peroxynitrite nor 3-NT showed statistically significant differences between any of the experimental groups (**Figure 8**).

### 3.8 Apoptosis markers

**Figure 9:**
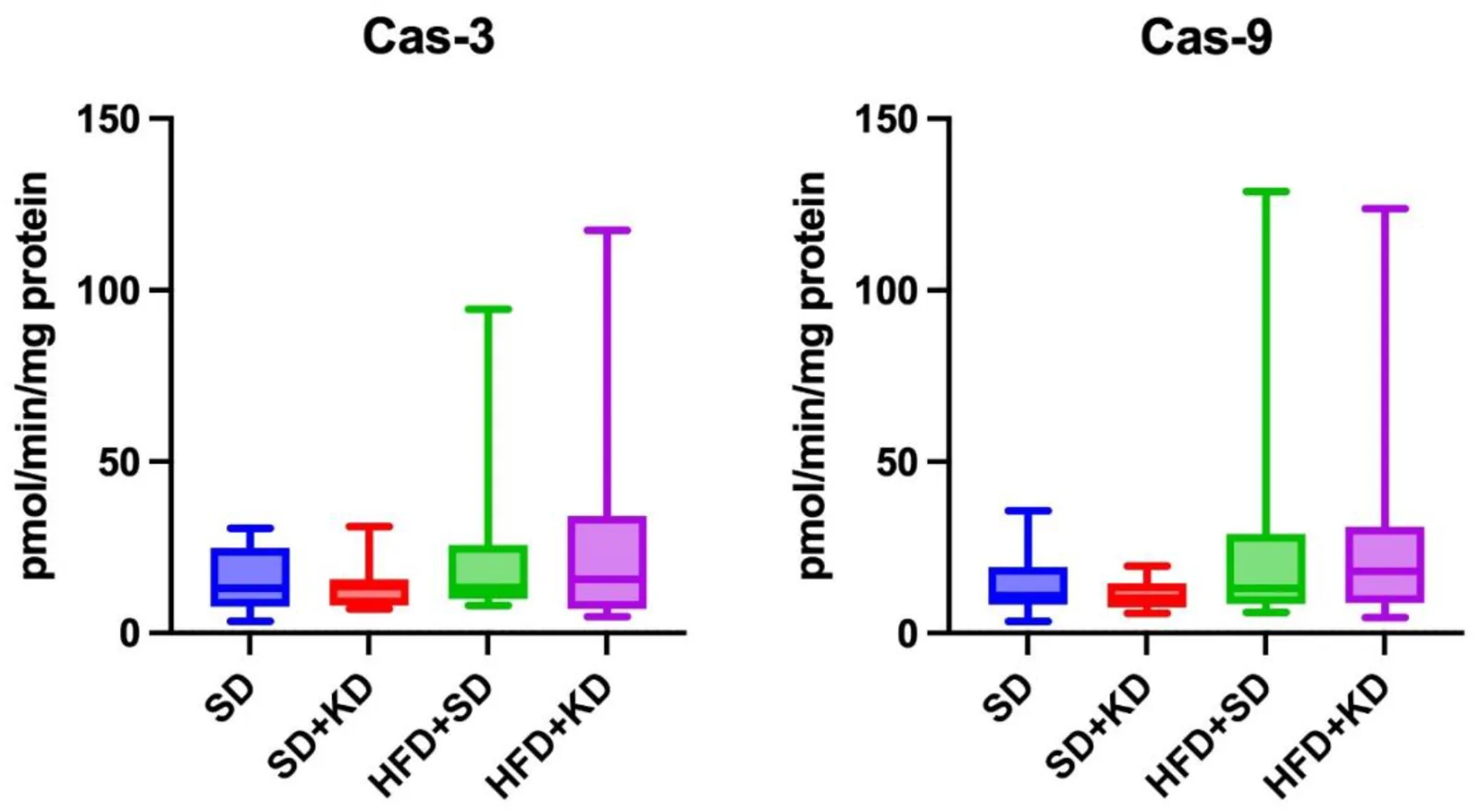
Concentration of apoptosis markers in the skin of rats. The results are presented as the median, minimum, maximum, and percentiles. CAS-3, caspase-3; CAS-9, caspase-9; HFD, high-fat diet; KD, ketogenic diet; SD, standard diet.

Caspase activity remained comparable across all dietary groups. Measurements of both Cas-3 and Cas-9 showed no statistically significant differences (**Figure 9**).

### 3.9 Signaling pathways associated with oxidative stress and inflammation

**Figure 10:**
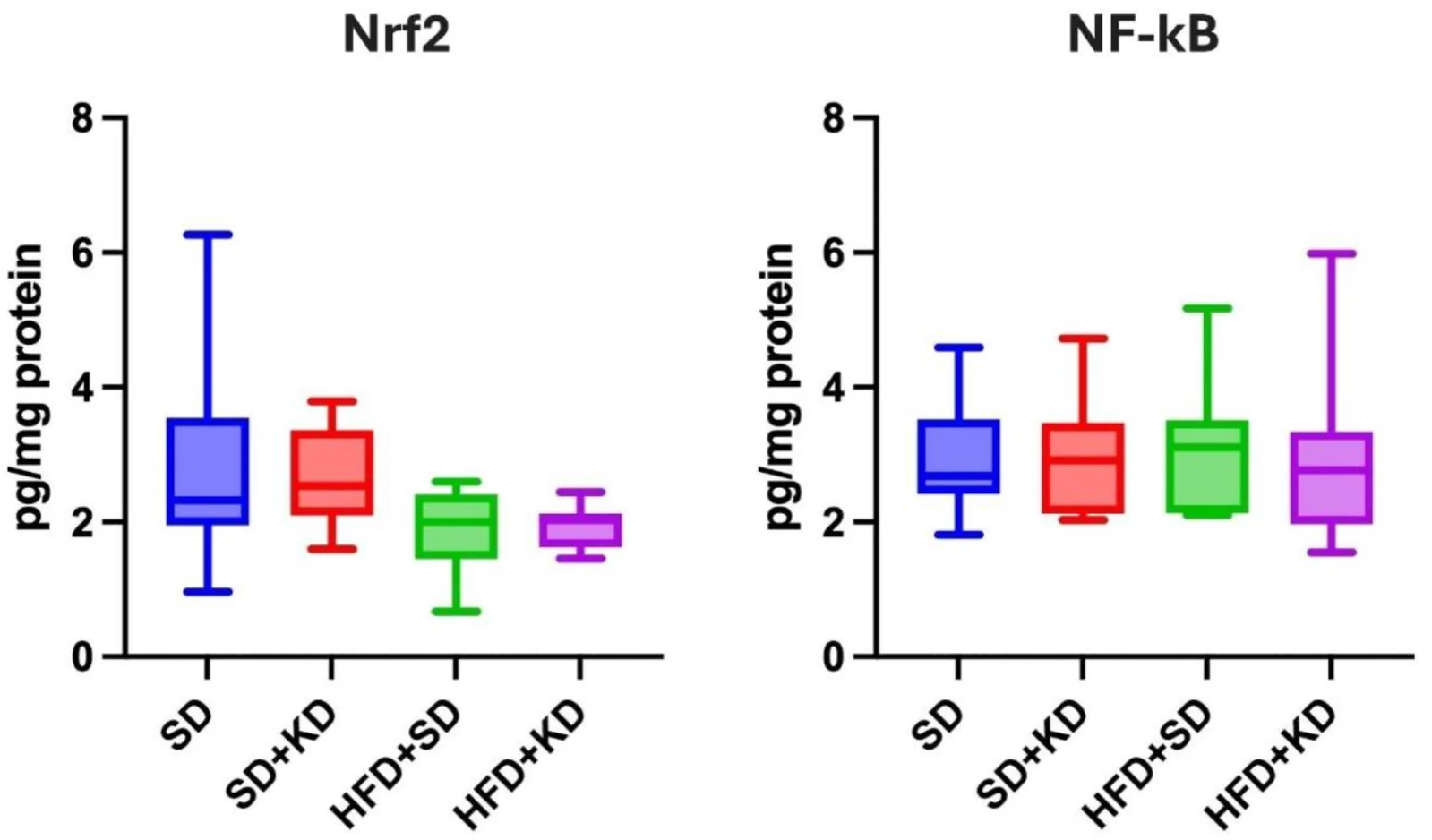
Expression of Nrf2 and NF-κB in the skin of rats. The results are presented as the median, minimum, maximum, and percentiles. HFD, high-fat diet; KD: ketogenic diet; NF-κB, nuclear factor kappa B; Nrf2, nuclear factor erythroid 2-related factor 2; SD, standard diet.

The expression levels of signaling proteins - Nrf2 and NF-κB - did not differ significantly among the four experimental groups (SD, SD+KD, HFD+SD, HFD+KD). Neither ketogenic diet nor prior exposure to high-fat feeding resulted in detectable alterations in Nrf2- or NF-κB-related signaling in the skin (**Figure 10**).

**Figure 11:**
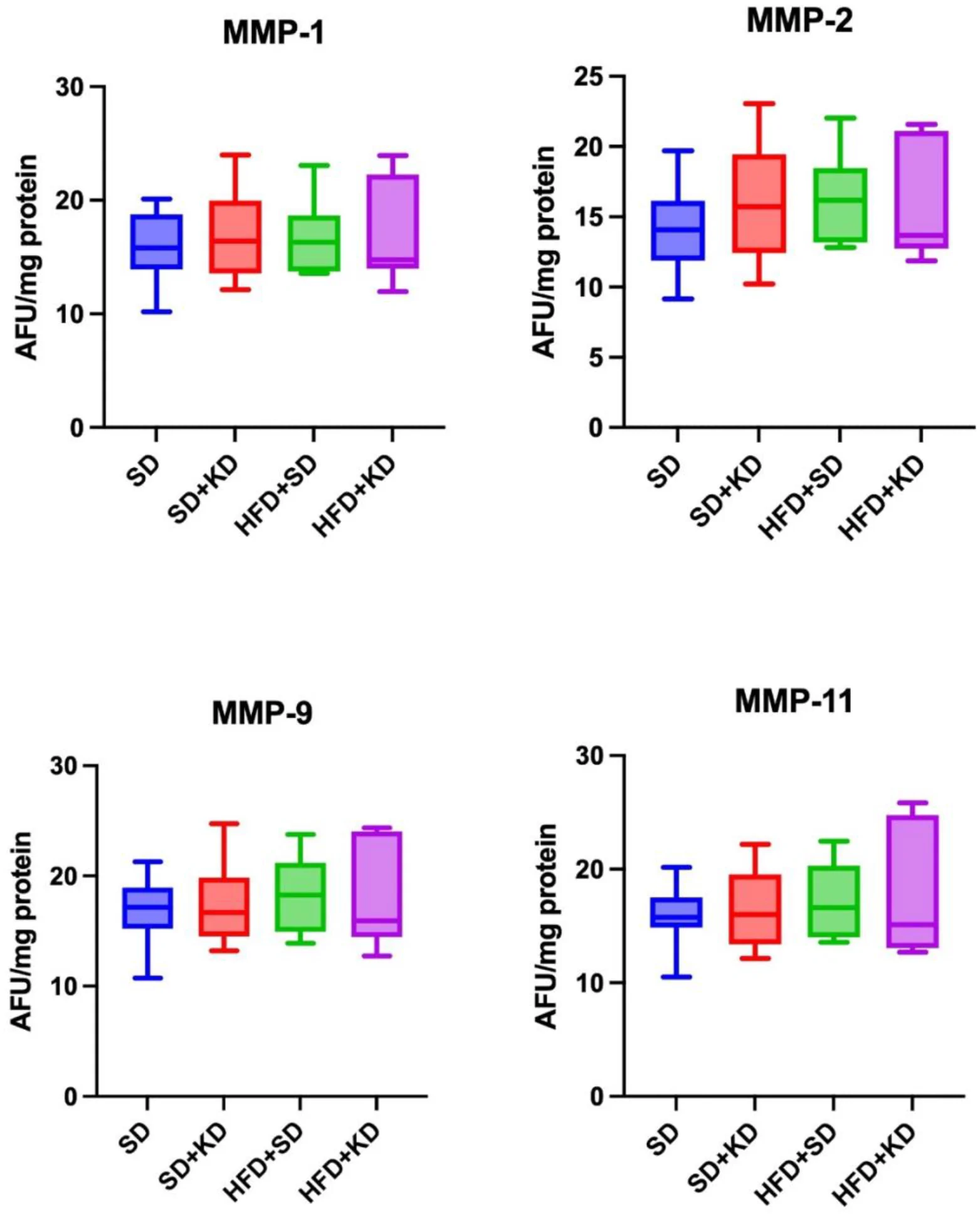
Expression of matrix metalloproteinases (MMP-1, MMP-2, MMP-9 and MMP-11) in the skin of rats. The results are presented as the median, minimum, maximum, and percentile. HFD, high-fat diet; KD, ketogenic diet; SD, standard diet; MMP, matrix metalloproteinase.

Similarly, no statistically significant differences were observed in the expression of matrix metalloproteinases between the experimental groups. The levels of all analyzed MMPs remained comparable across SD, SD+KD, HFD+SD, and HFD+KD animals (**Figure 11**).

## 4 Discussion

Obesity is a chronic metabolic disease that negatively impacts not only internal organs but also the skin (76). Adipokines produced by adipose tissue enhance the response of T helper lymphocytes type 1 and 17 (Th1/Th17) and activate the NF-κB inflammatory pathway (77). Consequently, keratinocyte proliferation is enhanced, accompanied by increased neutrophil recruitment and the production of ROS and RNS (78). Previous studies have shown that the skin of obese animals is characterized by increased oxidative stress, enhanced protein glycation, and elevated MMP expression, reflecting molecular alterations similar to those observed during skin ageing (79–82). In particular, HFD feeding has been shown to increase oxidative stress in rat skin, as evidenced by elevated levels of lipid peroxidation and protein oxidation markers, accompanied by alterations in antioxidant enzyme activity (83). Obesity has also been associated with increased accumulation of glycation and glycoxidation products in skin proteins. In obese Zucker rats, the formation of AGE/advanced lipoxidation end products (ALEs) in skin collagen was significantly higher than in lean animals, indicating that obesity may promote protein modification in the skin even in the absence of overt hyperglycemia (84). Taken together, these findings support the concept that obesity-related metabolic disturbances may contribute to oxidative and non-enzymatic modifications of skin proteins and may promote molecular alterations associated with skin ageing.

Nowadays, the KD is increasingly used in individuals with obesity not only to promote weight loss but also to improve skin health (26). However, its effects on the skin remain poorly understood. Studies investigating the effects of the KD on the skin are scarce and have been limited to models of inflammation and wound healing (85, 86). Importantly, no studies have evaluated the effects of the KD on skin metabolism in experimental models of obesity. To the best of our knowledge, this is the first study to investigate the effects of replacing the HFD with the KD on selected markers of oxidative stress, nitrosative stress, protein glycation, and ECM remodeling in rat skin. To address this research question, male Wistar rats were randomly assigned to four dietary groups: SD for 14 weeks, SD followed by KD, HFD followed by SD, or HFD followed by KD. Thus, after 8 weeks of initial feeding, the HFD was replaced with either the SD or KD, allowing us to assess the effects of dietary switching on skin metabolism. At week 8, obesity and hyperglycemia were confirmed in the HFD-fed animals. After 14 weeks, samples of shaved dorsal skin were collected for biochemical analyses. The absence of significant differences in most assessed parameters between the groups following dietary switching may indicate a potential protective effect of replacing the HFD with the KD or SD.

At the time of the dietary intervention (week 8), rats fed the HFD exhibited significantly higher body weight and fasting blood glucose levels than the control animals, confirming the successful induction of obesity and hyperglycemia. Following replacement of the HFD with either the KD or the SD, final body weight, blood glucose concentrations, and HOMA-IR were generally comparable across all groups. Similarly, no significant differences were observed in TAG and total cholesterol concentrations or liver enzyme activity, suggesting that replacing the HFD with either the KD or the SD exerts beneficial effects on the metabolic status of the rats. An increase in β-hydroxybutyrate concentration was observed in both groups receiving the KD, confirming the induction of a mild state of ketosis. These findings are consistent with the available evidence in the literature. Nasser et al. demonstrated that both a KD and a chow diet promoted weight loss after HFD feeding, although glycemic normalization was more pronounced in the KD group (87). Drabińska et al. demonstrated that a restrictive KD, as well as SD, slowed body weight gain in young Wistar rats with HFD-induced obesity, while final body weight remained comparable between groups at the end of the experiment (88). However, dietary-switch studies also indicate that not all HFD-induced abnormalities are fully reversible despite dietary normalization (89, 90). This is also confirmed by the results of our study.

KD has been shown to reduce oxidative stress and inflammation by reducing MDA and inflammatory markers such as C-reactive protein (CRP) and increasing serum TAC (91). One of the key mechanisms responsible for reducing oxidative stress during KD is the activation of the Nrf2 pathway (38). The Nrf2 transcription factor has been proven to be activated during metabolic adaptation to KD in response to increased levels of ROS (H_2_O_2_) and lipid peroxidation products, including 4-HNE (38). However, most of the evidence supporting this mechanism comes from studies investigating the effects of the KD on the liver, kidneys, and brain (41–43). In our study, expression of Nrf2 as well as the concentration and activity of cutaneous antioxidants did not differ between groups, with the exception of SOD activity, which was significantly higher in the HFD+SD group compared to SD and SD+KD. The increased scavenging of superoxide anion radicals by SOD may indicate an enhanced antioxidant response following prior exposure to an HFD. It is well known that O_2_^-^ is produced not only in the mitochondrial respiratory chain but also by the action of numerous enzymes (NADPH oxidase, xanthine oxidase), whose activity is enhanced by HFD (92–94). Excessive fat intake increases β-oxidation of fatty acids in mitochondria, which leads to the formation of reduced forms of coenzymes such as nicotinamide adenine dinucleotide (NADH) and flavin adenine dinucleotide (FADH_2_) (95, 96). These molecules supply electrons to the mitochondrial respiratory chain (96) and increase electron flow through the electron transport chain, which can lead to its overload and the overproduction of ROS (95–97). Studies have also shown that HFD increases ROS production by inducing NADPH oxidase, resulting in the activation of the NF-κB pathway, which plays a key role in inflammation (98). In our study, NF-κB expression did not differ significantly among the experimental groups. Total antioxidant capacity (TAC), total oxidant status (TOS), markers of lipid peroxidation (MDA and 4-HNE), and markers of protein oxidation (TT, PC and 3-NT) were also comparable across the groups. These findings suggest that replacing the HFD with the KD may restore physiological redox homeostasis and attenuate the oxidative stress associated with obesity and hyperglycemia. The effects of the KD on redox homeostasis appear to be comparable to those of the SD. Previous studies have shown that replacing an HFD with an SD can reverse several HFD-induced metabolic disturbances and restore physiological redox homeostasis (99–101). Specifically, these changes were accompanied by reductions in body weight and adipose tissue mass, as well as restoration of hepatic redox homeostasis, reflected by decreased oxidative damage, including lower levels of thiobarbituric acid-reactive substances (TBARS) and protein carbonyls (99). However, another data on the effects of the KD on oxidative metabolism in the skin are currently lacking.

Metabolic disturbances observed in obesity are closely associated with increased protein glycation (10, 102). Glycation is a non-enzymatic process occurring as a result of the Maillard reaction, in which reducing sugars react with the amino groups of proteins, lipids, and nucleic acids (20). AGEs cause the formation of cross-links in collagen, resulting in reduced skin elasticity and wrinkle formation (20, 103). Furthermore, they increase oxidative stress by reducing SOD activity and lead to increased production of metalloproteinases (MMPs), particularly MMP-1, MMP-2, and MMP-9, which degrade the extracellular matrix (20). Previous studies have shown that the formation of AGE/ALEs in skin collagen was two- to threefold higher in obese rats than in lean animals, suggesting that obesity may promote the accumulation of protein modifications in the skin (84). In our study, the HFD+SD group showed significantly higher levels of protein glycation products (CRO and AGE) and protein glycoxidation products (KN). These changes were not observed in the HFD+KD group, which potentially suggests that glycation-related modifications persist despite the replacement of the HFD with the SD. It should be emphasized that protein glycation is a long-term process; therefore, the persistence of glycation products in the HFD+SD group may reflect metabolic alterations induced during prolonged HFD feeding that were not fully reversed by switching to the SD (104, 105). AGEs are irreversible glycation products that accumulate over time and exhibit the ability to persist in tissues for a prolonged period (104, 105). Accumulation of glycated proteins is considered one of the most important mechanisms of skin aging (106, 107). Yet, data regarding the effect of KD on protein glycation remain scarce. Drabińska et al. (75) did not observe significant changes in AGE levels following KD administration in rats with HFD-induced obesity, but these studies included other tissues, such as liver, muscle, and plasma (75).

Moreover, we evaluated expression of dermal MMPs, which participate in the degradation of ECM components, such as collagen types I and III and elastin (103, 108). During the aging process, increased expression of MMPs occurs, leading to an imbalance between the synthesis and degradation of the dermal matrix, resulting in a loss of skin firmness and elasticity and the development of wrinkles (103). Oxidative stress is a key factor inducing this imbalance (107). ROS activate signaling pathways in dermal fibroblasts, including NF-κB, which increases the activity of MMPs, particularly MMP-1 and MMP-3 (107),. Activation of the mitogen-activated protein kinase (MAPK) pathway leads to the induction of activator protein 1 (AP-1), which increases MMP expression and inhibits transforming growth factor-β (TGF-β) signaling, further increasing collagen degradation in the skin (107). These alterations have also been reported in obesity (4, 109, 110). However, no significant differences in MMP (MMP-1, MMP-2, MMP-9, MMP-11) activity were observed in the present study, which is consistent with the absence of significant changes in NF-κB expression and most markers of oxidative stress. Therefore, our findings suggest that a 6-week dietary intervention may improve systemic metabolic homeostasis and contribute to the regulation of processes involved in ECM remodeling. Although an 8-week HFD feeding period is a commonly used model for inducing fully developed obesity and associated metabolic disturbances (49, 50), it should be borne in mind that this duration may have been too short to detect measurable changes in MMP expression or activity. ECM remodeling is a complex and tightly regulated process that depends on the balance between ECM synthesis and degradation. Further studies are needed to confirm our hypothesis and determine whether longer dietary interventions result in measurable changes in MMP expression and activity.

Experimental studies have shown that HFD may promote apoptosis through mechanisms involving oxidative stress, c-Jun N-terminal kinase (JNK) activation, and increased expression of the pro-apoptotic Bcl-2-associated X protein (Bax), accompanied by increased Cas-3 activation (111). HFD-induced obesity has also been associated with mitochondrial dysfunction, oxidative stress, and endoplasmic reticulum (ER) stress, which may contribute to the activation of apoptotic pathways (112). In our study, the activities of Cas-3 and Cas-9, as well as ONOO^−^ production and markers of oxidative stress, did not differ significantly among the experimental groups. These findings suggest that the dietary interventions may help maintain apoptotic homeostasis by attenuating the oxidative and nitrosative stress associated with obesity. However, it should be noted that our experimental design did not include a pair-fed control group or a control group receiving an isocaloric diet. Therefore, these findings should be interpreted with caution, and the potential protective effects of dietary intervention on apoptotic processes require further investigation. Consistent with our findings, Irfannuddin et al. demonstrated that KD did not significantly alter the number of Cas-3-positive cells in the hippocampal dentate gyrus of healthy rats (113).

Scientific reports confirm that KD may have beneficial effects on skin health, partially through its anti-inflammatory and antioxidant properties (26, 39, 114). Nevertheless, the mechanisms of KD’s action on the skin are not yet fully understood, and the number of available studies in this area remains limited (26). The literature emphasizes that KD potentially supports the treatment of certain inflammatory skin conditions, including psoriasis, acne, and hidradenitis suppurativa (26, 39, 44, 115). These conditions are closely associated with chronic inflammation, oxidative stress, and increased body weight (116, 117). It has been suggested that KD’s effect on the skin results from its modulating effects on systemic mechanisms (26). Numerous studies have demonstrated that the KD is an effective strategy for reducing body weight, thereby contributing to the alleviation of inflammatory skin diseases (118–121). Consistent with these findings, our results also suggest that the beneficial effects of switching from an HFD to a KD on body weight reduction and metabolic status may be related to the observed profile of oxidative stress, protein glycation, and ECM remodeling in rat skin. However, further studies are needed to determine the effects of this dietary intervention on skin metabolism (26, 39).

## 5 Strengths and limitations

The strengths of the present study should also be emphasized. To the best of our knowledge, this is the first study to investigate the effects of switching from an HFD to a KD or SD on skin-related outcomes. We evaluated a broad panel of enzymatic and non-enzymatic antioxidants, markers of oxidative and nitrosative stress, protein glycation and glycoxidation, as well as apoptosis in skin homogenates. In addition, a range of systemic parameters, including metabolic status and liver function, was assessed. Thus, our study provides new insights into the potential benefits of KD in individuals with obesity and provides a basis for further research into the effects of dietary interventions on skin metabolism and function.

Several important limitations of the study should also be acknowledged. In particular, the absence of a pair-fed or isocaloric control group precludes determining whether the observed changes resulted specifically from switching from the HFD to the KD or were attributable to reduced energy intake, weight loss, or withdrawal of the HFD. In addition, only male Wistar rats were included in the study; therefore, the findings cannot be directly extrapolated to female rats, and potential sex-related differences warrant further investigation. The use of an animal model may not fully reflect the metabolic, redox, and inflammatory mechanisms occurring in the human body. Moreover, assessing only selected biochemical parameters and lacking molecular analyses may prevent capturing the complexity of the processes being analyzed in skin tissue, especially if it is exposed to various environmental factors. Furthermore, no histological evaluation of the skin was performed, which limits the relationship between the observed biochemical changes and structural alterations in the tissue. Further studies, both molecular and *in vitro*, are necessary to mechanistically explain the effect of the diet on skin metabolism in obesity.

## 6 Conclusions

In summary, replacing the HFD with the KD normalizes body weight and blood glucose levels and may exert beneficial effects on skin metabolism by restoring redox homeostasis and physiological protein glycation and by modulating processes involved in ECM remodeling. Overall, the effects of the KD on skin metabolism appear to be comparable to those of the SD. However, unlike the KD, switching from the HFD to the SD did not fully prevent the accumulation of skin protein glycation and glycoxidation products. Further studies are needed to elucidate the long-term effects of the KD on skin metabolism and ECM remodeling in the context of metabolic diseases.

## Data Availability

The raw data supporting the conclusions of this article will be made available by the authors, without undue reservation.
